# Ion Current Rectification, Limiting and Overlimiting Conductances in Nanopores

**DOI:** 10.1371/journal.pone.0124171

**Published:** 2015-05-15

**Authors:** Liesbeth van Oeffelen, Willem Van Roy, Hosni Idrissi, Daniel Charlier, Liesbet Lagae, Gustaaf Borghs

**Affiliations:** 1 Research group of Microbiology, Vrije Universiteit Brussel, Brussels, Belgium; 2 IMEC, Leuven, Belgium; 3 Electron Microscopy for Materials Science (EMAT), Department of Physics, University of Antwerp, Antwerp, Belgium; 4 Institute of Mechanics, Materials and Civil Engineering, Université catholique de Louvain, Louvain-la-Neuve, Belgium; 5 Department of Physics, Katholieke Universiteit Leuven, Leuven, Belgium; Universidad de Castilla-La Mancha, SPAIN

## Abstract

Previous reports on Poisson-Nernst-Planck (PNP) simulations of solid-state nanopores have focused on steady state behaviour under simplified boundary conditions. These are Neumann boundary conditions for the voltage at the pore walls, and in some cases also Donnan equilibrium boundary conditions for concentrations and voltages at both entrances of the nanopore. In this paper, we report time-dependent and steady state PNP simulations under less restrictive boundary conditions, including Neumann boundary conditions applied throughout the membrane relatively far away from the nanopore. We simulated ion currents through cylindrical and conical nanopores with several surface charge configurations, studying the spatial and temporal dependence of the currents contributed by each ion species. This revealed that, due to slow co-diffusion of oppositely charged ions, steady state is generally not reached in simulations or in practice. Furthermore, it is shown that ion concentration polarization is responsible for the observed limiting conductances and ion current rectification in nanopores with asymmetric surface charges or shapes. Hence, after more than a decade of collective research attempting to understand the nature of ion current rectification in solid-state nanopores, a relatively intuitive model is retrieved. Moreover, we measured and simulated current-voltage characteristics of rectifying silicon nitride nanopores presenting overlimiting conductances. The similarity between measurement and simulation shows that overlimiting conductances can result from the increased conductance of the electric double-layer at the membrane surface at the depletion side due to voltage-induced polarization charges. The MATLAB source code of the simulation software is available via the website http://micr.vub.ac.be.

## Introduction

The voltage in a nanopore or nanochannel has previously been modeled using Neumann boundary conditions at the pore or channel walls [1–7], *i.e.*, by setting the normal component of the electric field *V*_⊥_ equal to the surface charge density *σ*_*S*_ divided by the permittivity *ϵ* of the fluid inside the pore or channel: *V*_⊥_ = *σ*_*S*_/*ϵ*. One thereby implicitly assumes that the normal component of the electric field inside the membrane equals zero. This is a restrictive boundary condition as ions outside the pore at the membrane can be co-responsible for screening the surface charges within the pore, resulting in equipotential lines not perpendicular to the surface within the membrane. Furthermore, Donnan equilibrium boundary conditions are often used to set the concentrations and voltages at both entrances of the nanopore [1, 2, 4, 8, 9]. This may also form a restrictive boundary condition as the equilibrium approximation is only valid at relatively low bias voltages.

**Fig 1:**
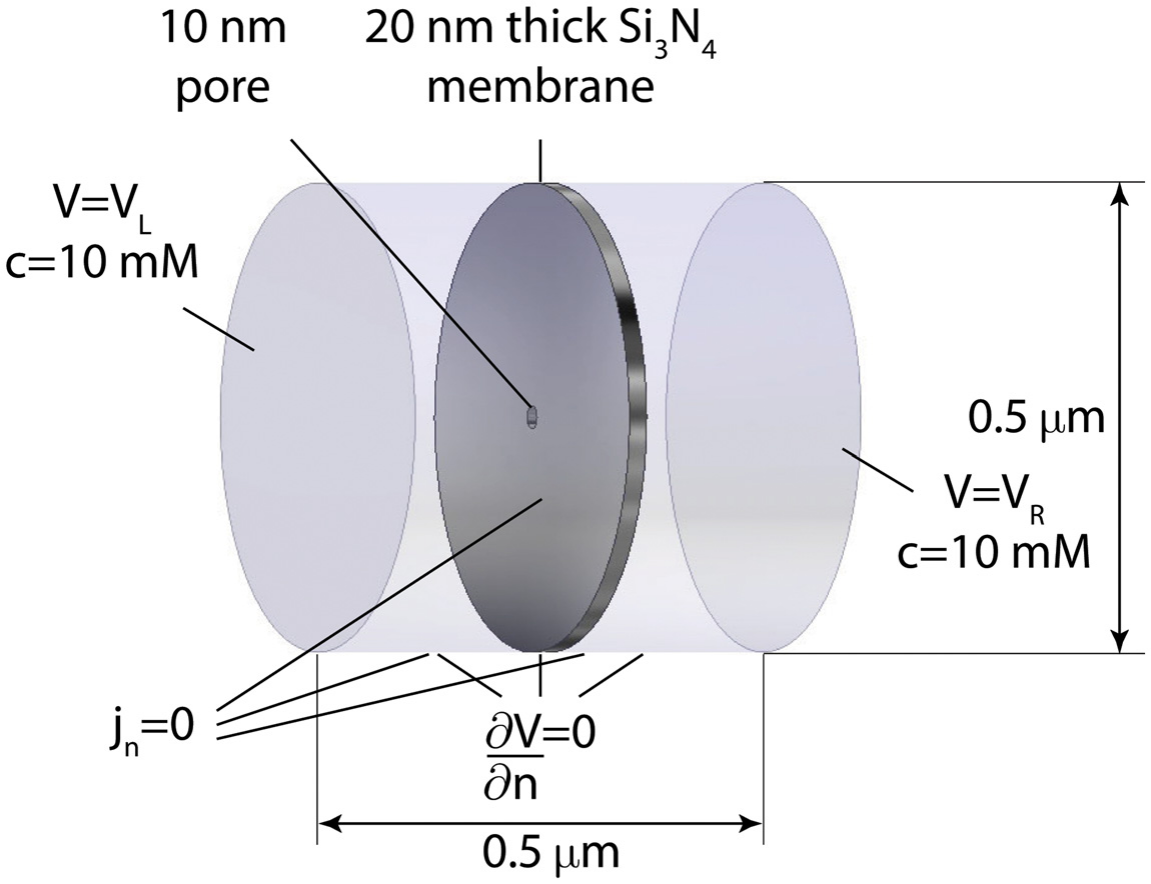
Geometry and boundary conditions of the simulations.

In this work, we show the effects of replacing these boundary conditions with less restrictive ones in time-dependent and steady state simulations. As model systems, we consider cylindrical and conical nanopores with a 10 nm inner diameter in 20 nm thick Si_3_N_4_ membranes with different surface charge geometries and immersed in a 10 mM KCl solution. The given values have mainly been chosen to yield a realistic nanopore with informative visualizations of concentrations and voltages: the Debye length is in the same order of magnitude as the pore size and membrane thickness. The boundary conditions are depicted in Fig 1. As concentration boundary conditions, we use *c* = 10mM at the left and the right, and set the ion fluxes through the remaining boundaries of the liquid equal to zero. As voltage boundary conditions, we apply *V*_*L*_ at the left and *V*_*R*_ at the right, and at the cylindrical boundary, we set the normal component of the electric field equal to zero. Hence, voltage boundary conditions are not applied at the membrane, but we do apply fixed surface charges there. These boundary conditions are indeed less restrictive: except for the flux boundary condition at the membrane and within the pore, all other boundary conditions are applied relatively far away from the nanopore, taking the access resistance into account. Moreover, the further away from the pore, the more the situation is reduced to a 1D problem, corresponding to normal derivatives equal to zero at the cylindrical boundary.

## Materials and Methods

### Simulation software

We have implemented simulation software that solves the Poisson, the Nernst-Planck and the time-dependent continuity equations in cylindrical coordinates, both time-dependently and in steady state. The three equations are given below:

$$\begin{array}{c} \nabla .\left(\varepsilon \nabla V\right)=-F\sum_{i}z_{i}c_{i} \end{array}$$ (1)

$$\begin{array}{c} j_{i}=-\left[D_{i}\left(\nabla c_{i}+c_{i}\frac{z_{i}e}{kT}\nabla V\right)\right] \end{array}$$ (2)

$$\begin{array}{c} \frac{\partial c_{i}}{\partial t}=-\nabla .j_{i}. \end{array}$$ (3)

The first equation is the Poisson equation, according to which the voltage *V* is calculated in a medium with permittivity *ϵ* and in the presence of ion species *i*, being K^+^ or Cl^−^ in this work. *F* is the Faraday constant, and *z*_*i*_ and *c*_*i*_ represent the charge number and concentration of ion species *i*, respectively. The second equation is the Nernst-Planck equation with *e* the elementary charge, **j**_**i**_ the flux of ion species *i* and *D*_*i*_ its diffusion coefficient: *D*(K^+^) = 1.95 × 10^−9^m^2^/s and *D*(Cl^−^) = 2.03 × 10^−9^m^2^/s. Substitution of the Nernst-Planck equation into the third equation, the continuity equation, allows the determination of concentration changes over time for each ion species *i*:

$$\begin{array}{c} \frac{\partial c_{i}}{\partial t}=-\nabla .\left[D_{i}\left(\nabla c_{i}+c_{i}\frac{z_{i}e}{kT}\nabla V\right)\right]. \end{array}$$ (4)

This equation will further be referred to as the ‘time-dependent Nernst-Planck equation’.

#### Time-dependent PNP simulations

Our simulation software considers two time steps: *δt*_*P*_ for the Poisson equation, and *δt*_*NP*_ for the time-dependent Nernst-Planck equation. *δt*_*P*_ is a fraction (*e.g.*, 1/40) of the time constant *τ* = *RC* = 1 × 10^−8^s corresponding to the resistance *R* = 16.7MΩ of the solution in series with the membrane capacitance *C* = 0.65fF. This time step is typically much larger than the time step *δt*_*NP*_ for the Nernst-Planck equation, which is a fraction (*e.g.*, 1/5) of Δ*x* ^2^/*D*, with Δ*x* the smallest mesh size, and *D* the diffusion coefficient of the most mobile ion species, being Cl^−^. Every time step *δt*_*P*_, the Poisson equation is solved using piecewise linear finite elements, and new piecewise constant concentrations are determined with the time-dependent Nernst-Planck equation in *δt*_*P*_/*δt*_*NP*_ time steps of *δt*_*NP*_. This is repeated for several RC time constants.

Note that voltage responses in these time simulations are not directly comparable to actual measurements, since only a small volume around the nanopore is simulated. Simulations of realistic voltage responses would require taking into account the contributions of the volumes in series and in parallel that are currently not simulated, including the chip that supports the membrane [10], as well as the electrode resistances and the amplifier response.

#### Steady state PNP simulations

To determine the steady state solution, we use the following strategy to accelerate the convergence of the time-dependent simulation: after a time-dependent simulation of several RC time constants, we solve the steady state Nernst-Planck equation for both K^+^ and Cl^−^, *i.e.*, by setting Eq 4 equal to zero. Subsequently, the average change in the concentrations relative to the end situation of the time simulation is calculated and added to both the K^+^ and Cl^−^ concentrations, thereby keeping the electric field unchanged, and a new time simulation is started. This process is repeated until the current contributions of both ion species through planes parallel with the membrane are spatially uniform up to 0.5% of the total current, or, when a zero voltage is applied, 0.5% of the estimated current at 0.1 V, determined from the pore and access resistances [11] with ion concentrations as in the solution. The tolerance of 0.5% was chosen to limit simulation times.

### Nanopore fabrication and measurements

Membranes were fabricated as described by Rosenstein *et al*. [12], although without local membrane thinning, and, similarly to Han *et al*. [13], the oxide underneath the membrane has been removed by KOH etching instead of BHF. We produced membranes with sizes varying from a few to tens of *μ*m and selected those smaller than 10 *μ*m as they are less fragile. These membranes were immersed in an aqueous solution containing 100 mM KCl, 20 mM Tris at pH = 8.0 and 0.5 mM EDTA, after which nanopores were formed with dielectric breakdown [14]. Current-voltage characteristics were measured using a custom-designed amplifier that, in contrast with the Axopatch amplifier, allowed us to apply bias voltages larger than 1 V.

## Results and Discussion

In this section, we first investigate current convergence over time. Subsequently, we study time-dependent and steady state simulations of a cylindrical nanopore in an uncharged membrane, the effects of surface charges occurring in different configurations, and those of a conical shape. We compare our simulations with measurements and previous reports.

### Current convergence toward steady state

**Fig 2:**
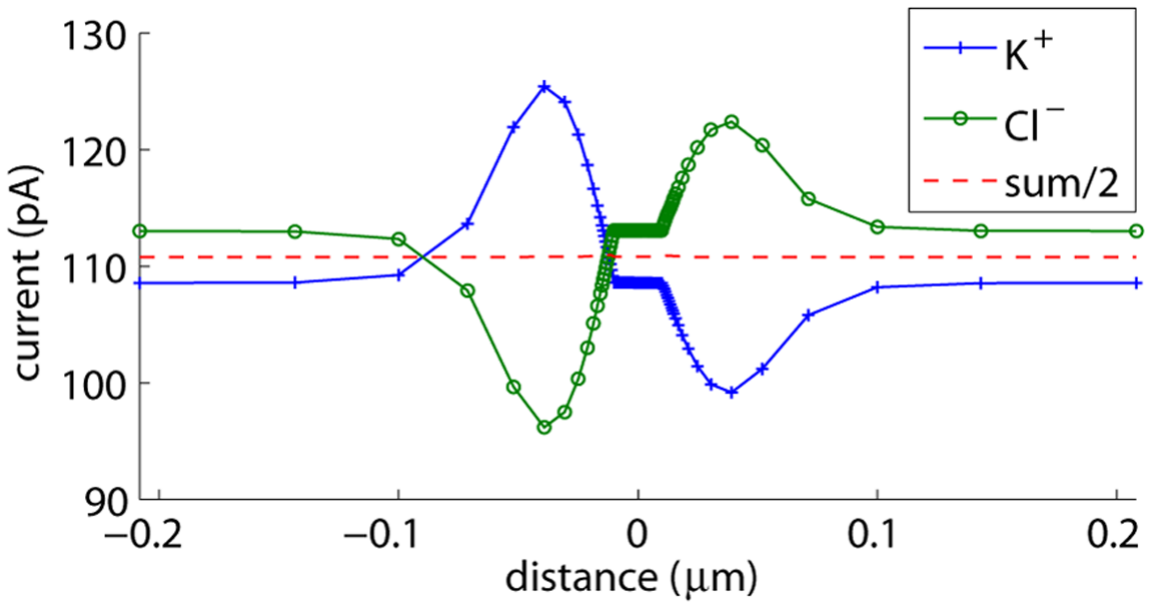
Currents carried by the K^+^ and the Cl^−^ ions for an uncharged cylindrical nanopore. Current contributions through planes parallel with the membrane are determined 0.2 *μ*s after applying a voltage step of *V*_*L*_−*V*_*R*_ = 0.5 V across the nanopore.

In our time simulations, we noticed that steady state could not be reached, even after 0.2 *μ*s, which corresponds to 20 RC time constants. For example, Fig 2 shows the currents carried by the K^+^ and the Cl^−^ ions 0.2 *μ*s after applying a voltage step of *V*_*L*_−*V*_*R*_ = 0.5 V across an uncharged membrane with a cylindrical pore. After 0.2 *μ*s, the membrane capacitor is almost completely charged, resulting in a zero polarization current inside the membrane, and therefore, the total ion current is spatially uniform. However, this is not true for the individual ion contributions, indicating that concentrations are still changing over time and therefore that steady state is not reached. With common mode and differential mode fluxes defined as **j**_**c**_ = (**j**_**K**^+^_+**j**_**Cl**^−^_)/2 and **j**_**d**_ = (**j**_**K**^+^_−**j**_**Cl**^−^_)/2 resp., individual ion fluxes can be expressed as **j**_**K**^+^_ = **j**_**c**_+**j**_**d**_ and **j**_**Cl**^−^_ = **j**_**c**_−**j**_**d**_. The differential mode flux corresponds to half of the total charge flux. After 20 RC time constants, the divergence of this flux is almost zero, indicating a situation that approaches electroneutrality. Therefore, it is the common mode flux that slows down convergence of the individual ion fluxes. This flux does not directly change the electric fields: both the K^+^ and the Cl^−^ concentrations change by the same amount *δc*, which does not affect the voltages determined with the Poisson equation. The effect of the common mode flux is rather indirect: concentration changes result in common mode and differential mode flux changes.

$$\begin{array}{c} \delta j_{c}=-\left(D_{K^{+}}+D_{{\mathrm{Cl}}^{-}}\right)/2\nabla \delta c-\left(D_{K^{+}}-D_{{\mathrm{Cl}}^{-}}\right)/2\delta c\frac{e}{kT}\nabla V \end{array}$$ (5)

$$\begin{array}{c} \delta j_{d}=-\left(D_{K^{+}}-D_{{\mathrm{Cl}}^{-}}\right)/2\nabla \delta c-\left(D_{K^{+}}+D_{{\mathrm{Cl}}^{-}}\right)/2\delta c\frac{e}{kT}\nabla V. \end{array}$$ (6)

The differential mode flux changes in a non-divergent way, preserving approximate electroneutrality. This allows the total current to change in response to resistance variations induced by concentration changes. When these resistance variations are small, for example when concentration changes are relatively small or occur outside the nanopore as in Fig 2, *δ* **j**_**d**_ ≈ 0, and thus,

$$\begin{array}{c} \delta j_{c}\approx -2\frac{D_{K^{+}}D_{{\mathrm{Cl}}^{-}}}{D_{K^{+}}+D_{{\mathrm{Cl}}^{-}}}\nabla \delta c. \end{array}$$ (7)

Hence, the convergence of the common mode flux is limited by co-diffusion: diffusion of both K^+^ and Cl^−^ with an intermediate diffusion coefficient: $D_{\text{int}}=2\frac{D_{{\text{K}}^{+}}D_{{\text{Cl}}^{-}}}{D_{{\text{K}}^{+}}+D_{{\text{Cl}}^{-}}}$. To reach the boundaries of the simulation domain at 250 nm from the membrane, this process would take about (250nm)^2^/*D*_int_ = 30*μ*s, while it would take about an hour in a realistic situation with millimeter-sized compartments. Hence, true steady state will not be reached in reality, even though the current may level off quite rapidly during a measurement or in a simulation.

In Fig 2, we see that the individual current contributions of K^+^ and Cl^−^ within the pore and further away from the membrane are determined by their diffusion coefficients. Close to the membrane, we additionally observe co-diffusion of K^+^ and Cl^−^ towards the membrane: the average concentration of K^+^ and Cl^−^ at the membrane increases by removing K^+^ and Cl^−^ ions from the bulk solution. This effect is not nanopore-related: it also takes place at a membrane without a nanopore. In that case, the time simulation will ultimately converge to the equilibrium situation in which the externally applied voltage induces polarization charges at the membrane surface, and the K^+^ and Cl^−^ concentrations resp. decrease and increase exponentially with voltage changes at the membrane. Hence, the average concentration at the membrane increases compared to the bulk concentration, requiring a common-mode flux and thus co-diffusion of both ion species towards the membrane.

In our simulations, the Neumann boundary conditions applied relatively far away from the pore set the equilibrium situation at these boundaries. Closer to the nanopore, the increase in average concentrations due to polarization charges positively affects the conductance of the electric double-layer at the membrane, and thus the nanopore conductance.

### Simulation of an uncharged nanopore

**Fig 3:**
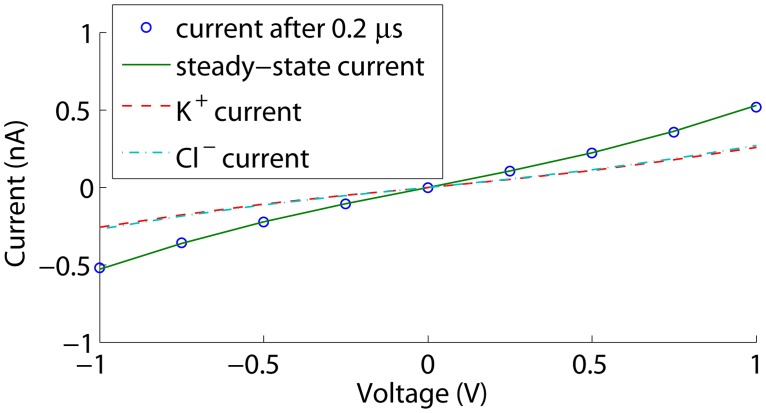
Currents through an uncharged cylindrical nanopore. The current after 0.2 *μ*s is shown together with the steady state current and the K^+^ and Cl^−^ contributions at steady state.

To assess whether the time-dependent simulations converge sufficiently to be representative for the measured steady currents, we also performed steady state simulations. The corresponding I-V curves are shown in Fig 3. The current increases in absolute value toward steady state as the double-layer conductance increases at the membrane, but the differences remain smaller than 2% in the considered voltage range. Furthermore, higher absolute voltage biases result in higher polarization charge densities on the membrane, and thus an increased conductance of the electric double-layer at the membrane. This explains the slight deviation from a linear current-voltage relation at larger absolute voltage biases.

**Fig 4:**
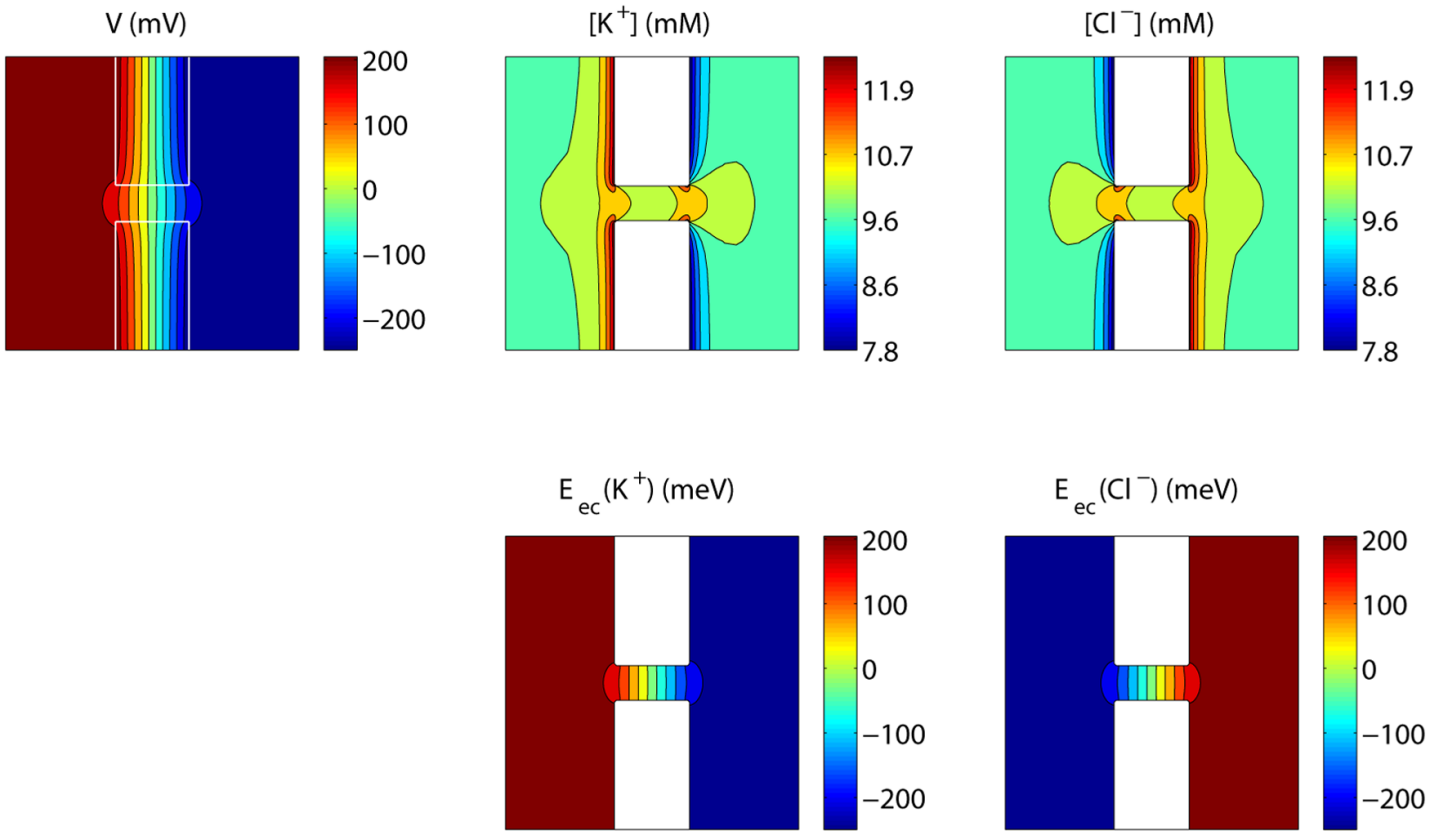
Steady state voltage, concentrations and electrochemical potentials for an uncharged nanopore. The values shown are those in a plane through the axis of the nanopore, with *V*_*L*_−*V*_*R*_ = 0.5 V.

Fig 4 shows the steady state voltage and concentration profiles corresponding to *V*_*L*_−*V*_*R*_ = 0.5 V in a plane through the axis of the nanopore, and the corresponding electrochemical potentials: $E_{\text{ec},i}=z_{i}qV+k_{B}T\text{ln}(\frac{c_{i}}{c_{Bi}})$, with *k*_*B*_ the Boltzmann constant, *T* the absolute temperature, and *c*_*Bi*_ the bulk concentration of ion species *i*. At steady state, electrochemical equipotential lines are perpendicular to the membrane surface and ions follow a tangential flow at this surface. In contrast, voltage equipotential lines are not perpendicular to the surface, showing indeed that the boundary condition *V*_⊥_ = *σ*_*S*_/*ϵ* at the nanopore surface would impose limitations.

### Influence of surface charges in a cylindrical nanopore

**Fig 5:**
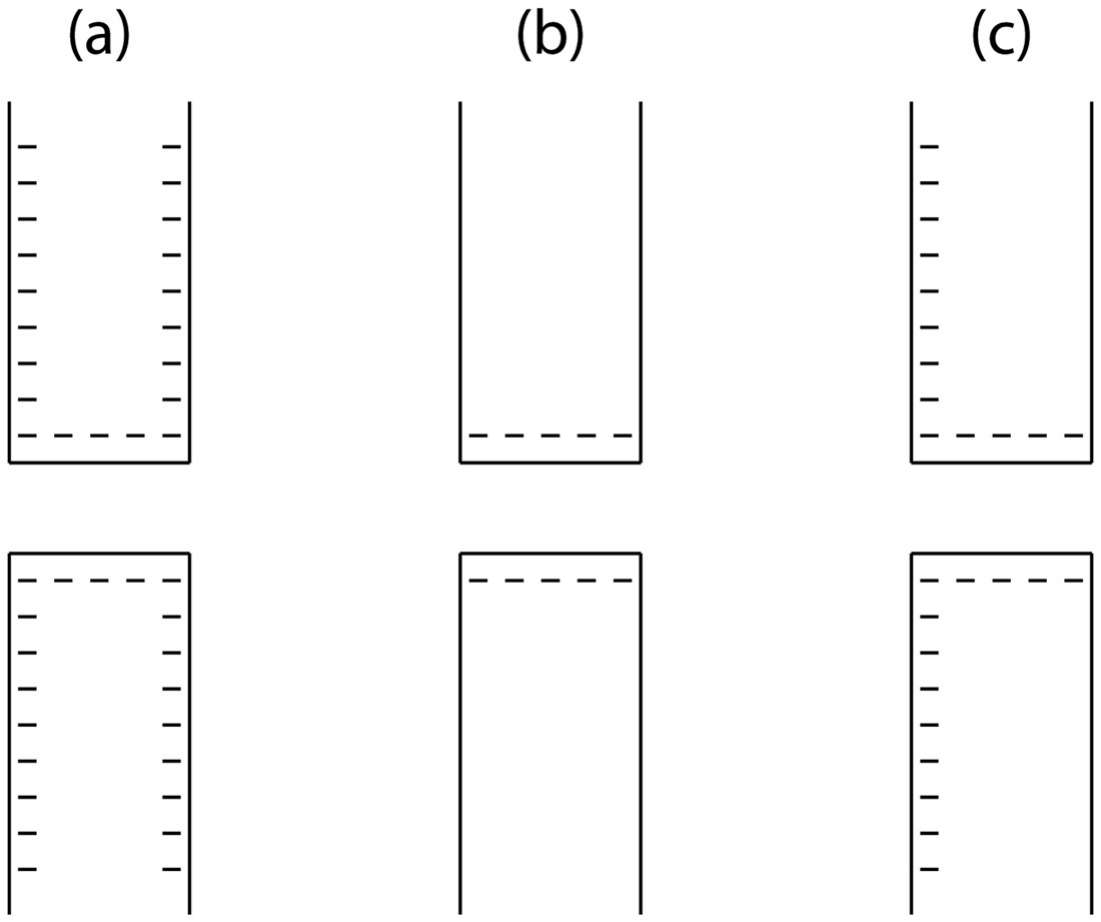
Simulated surface charge configurations.

We studied the influence of surface charges of −50mC/m^2^ appearing in the configurations shown in Fig 5. To initialize the concentrations, we applied an external voltage of 0 V and let the double layer build up using a steady state simulation. Then the voltage was changed to the desired value, and a time-dependent simulation was started.

#### Simulation of a uniformly charged nanopore

**Fig 6:**
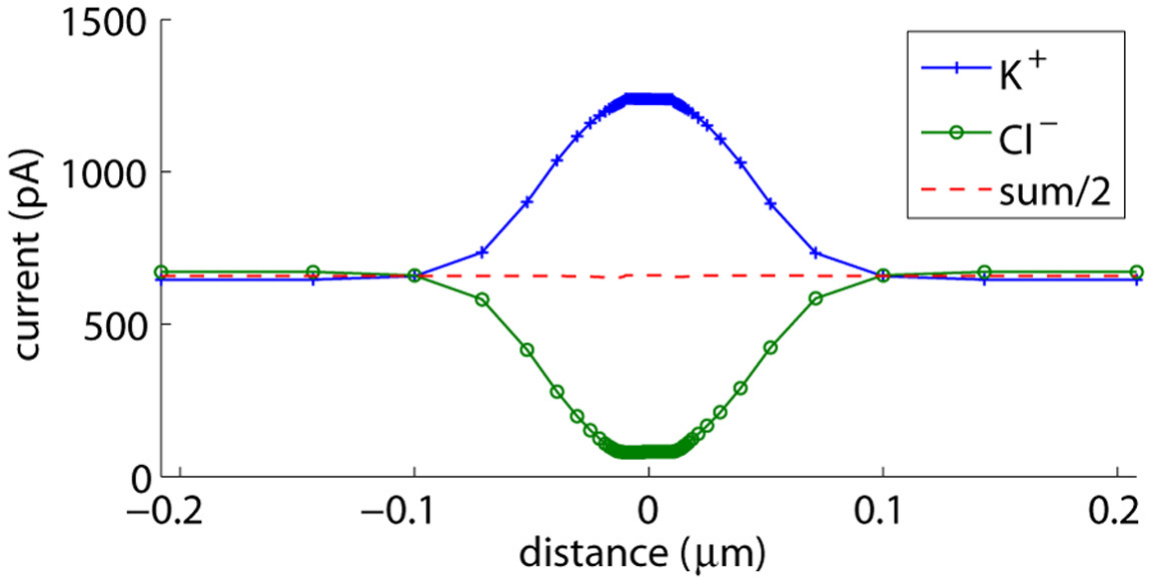
Currents carried by the K^+^ and the Cl^−^ ions for the nanopore in Fig 5a. Current contributions through planes parallel with the membrane are determined 0.2 *μ*s after applying a voltage step of *V*_*L*_−*V*_*R*_ = 0.5 V across the nanopore. The surface charge density is −50mC/m^2^.

Fig 6 shows the currents 0.2 *μ*s after applying a voltage step of *V*_*L*_−*V*_*R*_ = 0.5 V across the membrane with the configuration in Fig 5a. We observe virtually perfect ion selectivity within the nanopore but not within the bulk, in agreement with an almost complete depletion of Cl^−^ ions inside the nanopore. This causes ion concentration polarization [15], also called the ion-enrichment and ion-depletion effect [16]: both ion concentrations decrease at the left and increase at the right of the nanopore, giving rise to co-diffusion of both ion species respectively towards and away from the nanopore.

**Fig 7:**
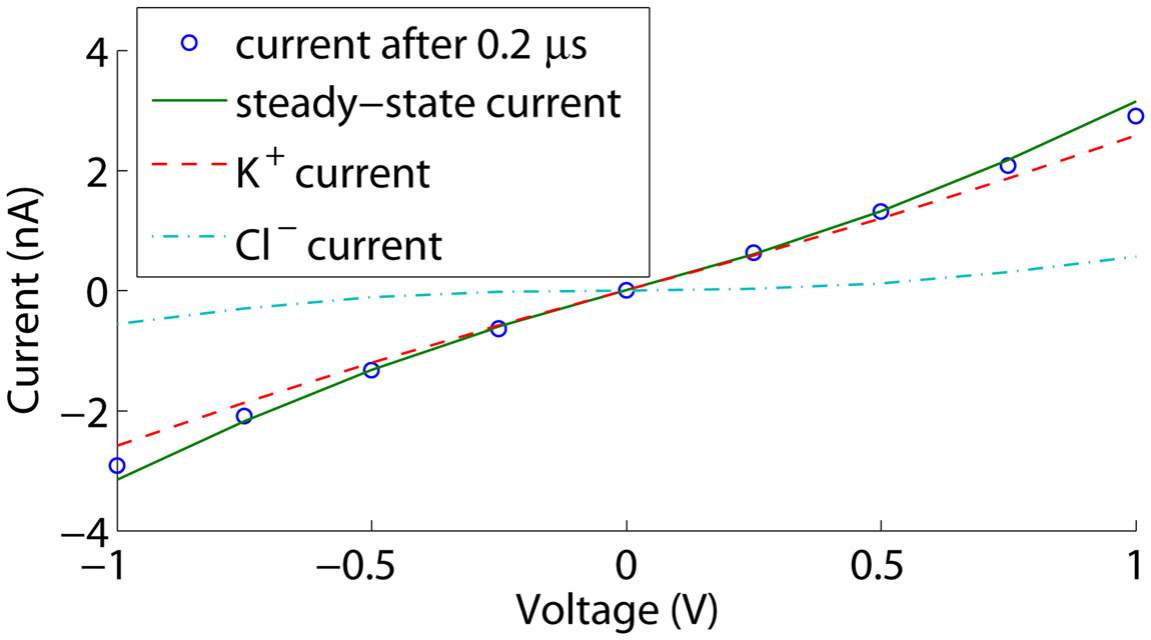
Currents through the nanopore in Fig 5a. The current after 0.2 *μ*s is shown together with the steady state current and the K^+^ and Cl^−^ contributions at steady state. The surface charge density is −50mC/m^2^.

As conductivities are proportional to concentrations, the conductivity at the left decreases, while it increases at the right. This results in a minor net conductance decrease, slowing down convergence towards the steady state current at voltages < 0.5 V. This effect is however hardly visible in Fig 7: at 0.25 V a decrease of only 4% is observed. At higher absolute voltage biases, the conductance decrease is compensated by an increase in conductance of the electric double-layer at the depletion side, due to negative polarization charges adding up to the fixed negative surface charges. This positive effect outweighs the negative effect at the accumulation side where positive polarization charges reduce the total surface charge and thus the electric double-layer conductance. Hence, a net positive effect on the conductance *G* = *I*/*V* is observed.

**Fig 8:**
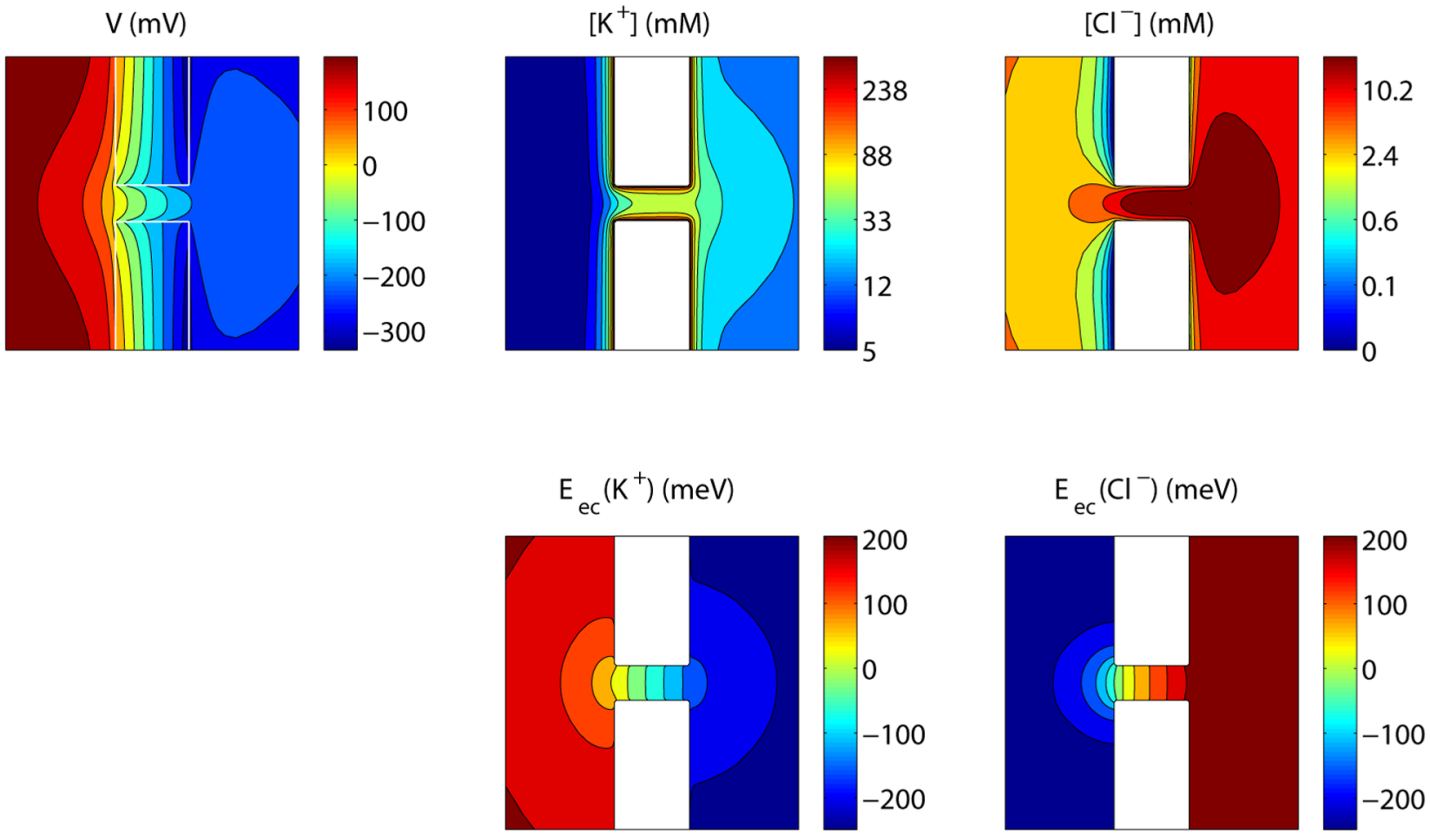
Steady state voltage, concentrations and electrochemical potentials for the nanopore in Fig 5a. The values shown are those in a plane through the axis of the nanopore, with *V*_*L*_−*V*_*R*_ = 0.5 V and a surface charge density of −50mC/m^2^. The fact that the electrochemical equipotential lines are not perfectly perpendicular to the membrane surface is an effect occurring at high electric fields due to the piecewise linear approximation in the voltage combined with piecewise constant concentrations. This effect is reduced by decreasing the mesh size, as shown in S1 Supporting Information.

Fig 8 shows the steady state voltage, concentrations and electrochemical potentials at an externally applied voltage of 0.5 V. The equipotential lines inside the pore are closer to each other for the Cl^−^ ions than the K^+^ ions, in agreement with the depletion of Cl^−^ in the pore, and the corresponding higher resistivity experienced by the Cl^−^ ions. Voltage equipotential lines are again not perpendicular to the nanopore surface, and we observe concentration polarization at both sides of the nanopore, with a slight concentration increase of Cl^−^ ions also extending within the pore.

#### Simulation of a charged nanopore in an uncharged membrane

**Fig 9:**
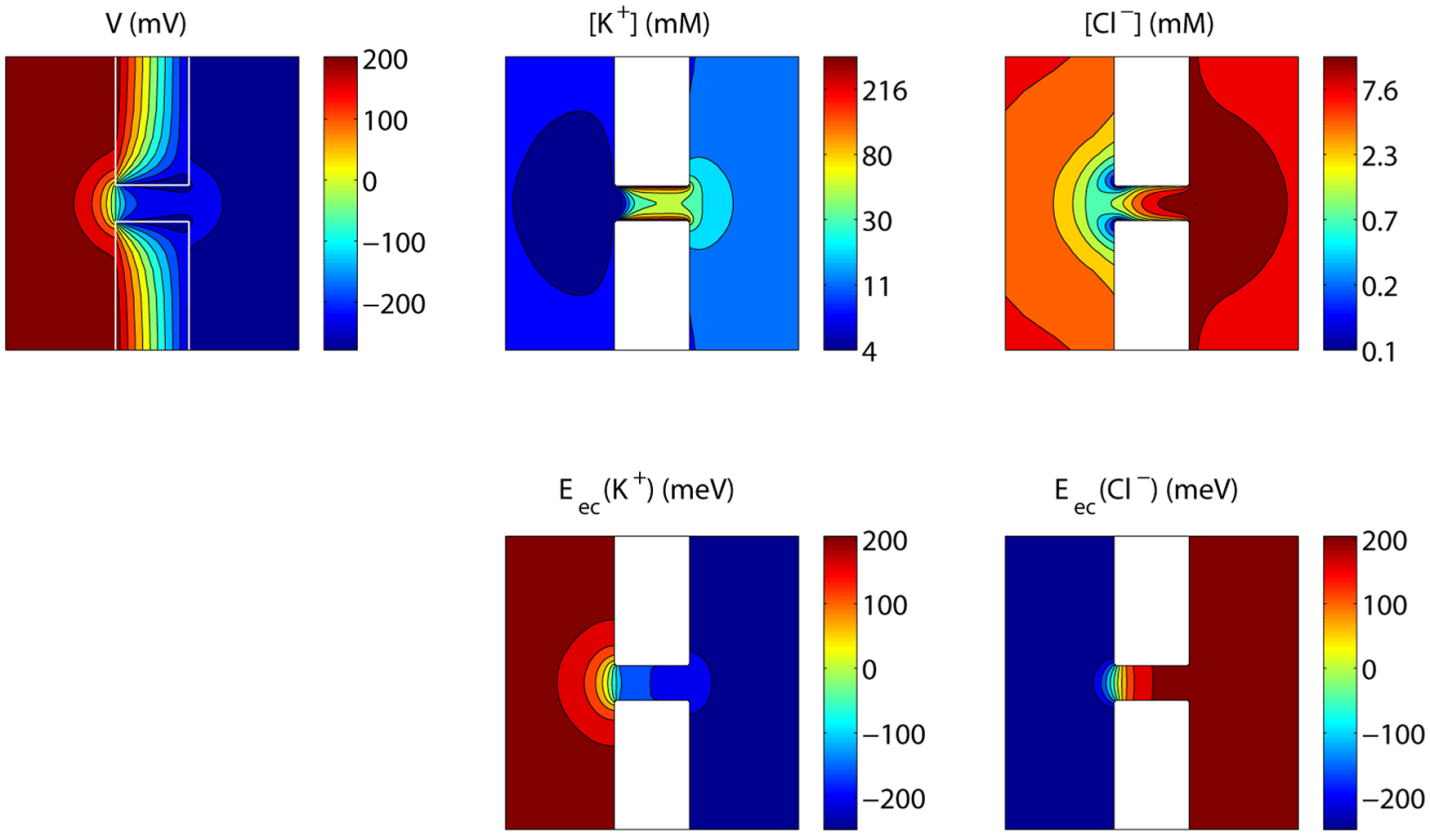
Steady state voltage, concentrations and electrochemical potentials for the nanopore in Fig 5b. The values shown are those in a plane through the axis of the nanopore, with *V*_*L*_−*V*_*R*_ = 0.5 V and a surface charge density of −50mC/m^2^.

**Fig 10:**
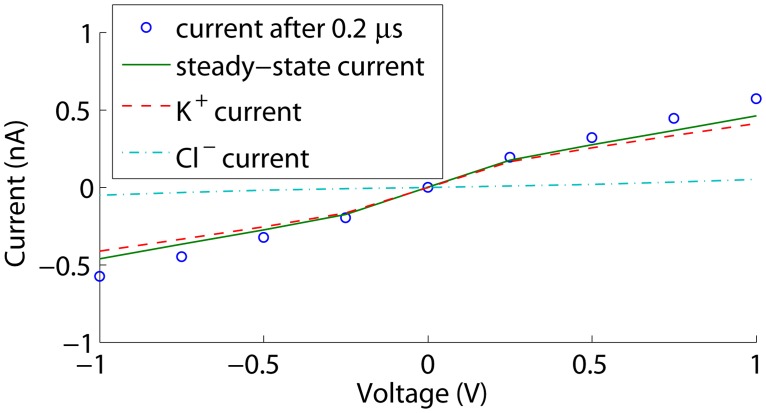
Currents through the nanopore in Fig 5b. The current after 0.2 *μ*s is shown together with the steady state current and the K^+^ and Cl^−^ contributions at steady state. The surface charge density is −50mC/m^2^.

In configuration b in Fig 5, the membrane is uncharged while the nanopore wall is charged. As in the previous case, the pore is selective, resulting in ion concentration polarization. Fig 9 shows the steady state voltage, concentrations and electrochemical potentials at an external voltage of 0.5 V. Both the K^+^ and Cl^−^ concentrations are decreased at the left of the pore, and increased at the right. The net effect is again a conductance decrease: the conductance of the depletion zone limits the current. Hence, this phenomenon corresponds to the ‘limiting current’ behaviour in nanoporous membranes and nanochannels [17]. As the depletion becomes more pronounced with increasing voltages, the conductance *g* = ∂*I*/∂*V* decreases, as observed in Fig 10. *g* seems to converge towards a constant value, corresponding to a situation where no additional concentration polarization occurs as the pore is not selective anymore over the additional current. Hence, ‘limiting conductance’ would be a more appropriate terms than ‘limiting current’, as the latter can only be obtained by perfect ion selectivity.

In contrast to the previous situation, the effect of the increase in conductance of the electric double-layer at the depletion side due to negative polarization charges is not visible. This is due to the lack of fixed surface charges, and the fact that the polarization charge density at 1V is only 3 mC/m^2^. Thus, within the considered voltage range, the conductance of the electric double-layer remains negligible compared to the double-layer conductance within the nanopore.

Even though ion concentration polarization and limiting currents are well-known effects in nanoporous membranes and nanochannels [15, 16], they seem to have come as a surprise when measuring gated single conical nanopores. In [18], the gate was actually positioned inside the nanopore instead of at the membrane surface, resulting in a situation similar to the configuration in Fig 5b, and thus a limiting conductance. Moreover, a further increase of the voltage in the limiting current regime has often led to the observation of overlimiting currents in nanochannels and nanoporous membranes: a state of higher conductance *g* = ∂*I*/∂*V* due to fluid dynamics, resulting in propagation and microvortices (see [15, 17, 19] and references therein). Fluid dynamics, and therefore the effect of electro-osmosis, is however outside the scope of the current manuscript. Modeling fluid dynamics would require the additional implementation of the Navier-Stokes equations. The effect of the omission is that actual currents may be underestimated, firsty, because fluid flow through the nanopore occurs mainly in the direction of the counterions dragging the fluid along, as almost no co-ions enter the pore. Secondly, the concentration decrease in the depletion zone may be limited due to microvortices, if these would occur in the case of a membrane with a single nanopore, leading to an overlimiting current. Note that, if an overlimiting current is observed in a thin membrane with a single nanopore, microvortices are not the only possible explanation: the increased conductance of the electric double-layer due to increased polarization charges at higher absolute voltage biases may also play a role.

#### Simulation of an asymetrically charged nanopore

**Fig 11:**
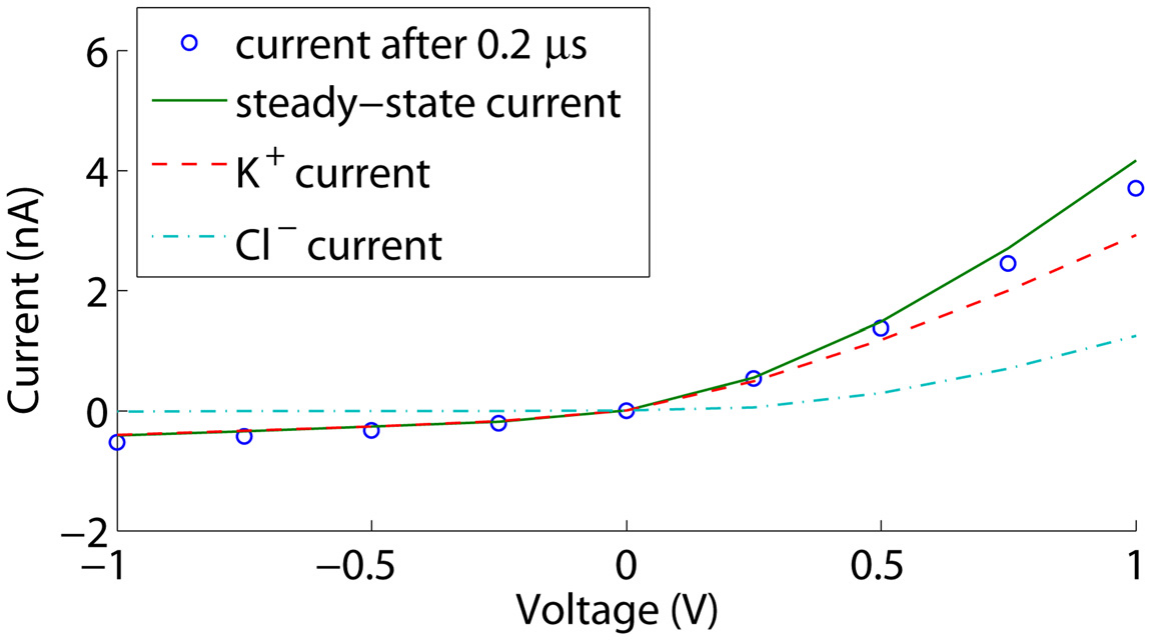
Currents through the nanopore in Fig 5c. The current after 0.2 *μ*s is shown together with the steady state current and the K^+^ and Cl^−^ contributions at steady state. The surface charge density is −50mC/m^2^.

**Fig 12:**
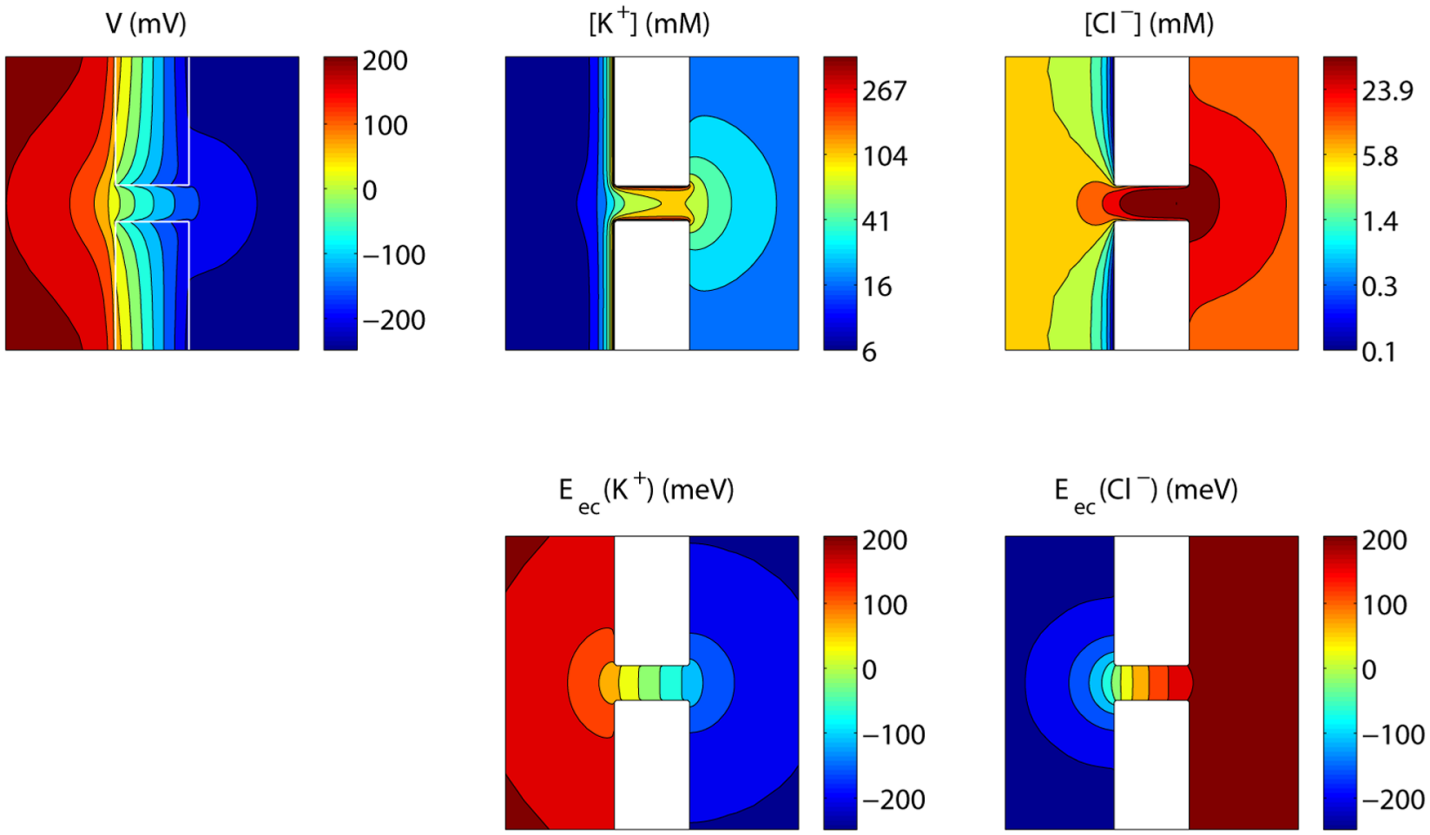
Steady state voltage, concentrations and electrochemical potentials for the nanopore in Fig 5c. The values shown are those in a plane through the axis of the nanopore, with *V*_*L*_−*V*_*R*_ = 0.5 V and a surface charge density of −50mC/m^2^.

With the asymmetric configuration in Fig 5c, ion current rectification is observed as shown in Fig 11. For positive voltages, concentration polarization occurs as a decrease of concentrations at the left of the nanopore where the electric double-layer conductance is high, and an increase of concentrations within the pore and at the right where the electric double-layer conductance is low, as displayed in Fig 12. This results in a net positive effect on the conductance. With increasing voltages, the difference in the currents carried by the counterions and the co-ions increases, concentration polarization becomes more pronounced, and therefore also its effect on the conductance *G*. However, the conductance decrease at the left gains importance compared to the conductance increase at the right, and ∂*g*/∂*V* decreases. *g* increases towards a constant value, where no additional concentration polarization occurs as again the pore is not selective anymore over the additional current.

Furthermore, the conductance may start increasing again due to a conductance increase of the electric double-layer at the depletion side, as the depletion region is similar to that of the configuration in Fig 5a. At negative voltages, the depletion region is similar to that of the configuration in Fig 5b, and a limiting conductance is observed that is not affected by double-layer concentration changes in the considered voltage range. Hence, the observed ion current rectification results from concentration polarization in combination with an asymmetric surface charge distribution, and may be positively affected by double-layer conductance variations due to the applied voltage bias.

In thick membranes, variations in polarization charge densities, and therefore double-layer conductance variations are negligible in a voltage range of several Volts. Hence, *g* reaches a constant value for both positive and negative voltages. As ∂*g*/∂*V* ≥ 0, the rectification ratio *G*(+*V*)/*G*(−*V*)∣_*V* > 0_ increases with *V* up to a constant value, as has been observed by Schiedt *et al*. [20] Moreover, as increased absolute surface charges within the nanopore lead to higher differences in the currents carried by the counterions and the co-ions, they also lead to increased effects on the conductance *G*, and therefore higher rectification ratios. Furthermore, it has experimentally been shown that the rectification ratio first increases with decreasing bulk concentrations, and then decreases again [20, 21]. This can be understood as follows: if the bulk concentrations are decreased, both the K^+^ and Cl^−^ currents decrease, but the difference between the two increases at first as the surface current of counterions does not decrease as fast. Concentration polarization is therefore enhanced, and the rectification ratio increased. When the concentrations are further decreased, the surface current decreases as well since a major part of the potential drop occurs outside the nanopore. The difference in the K^+^ and Cl^−^ currents decreases, concentration polarization is reduced and the rectification ratio decreases. At which concentration the surface current starts to decrease will depend on the membrane charges outside the nanopore.

As previously mentioned by Kubeil *et al*., the ratio of the pore radius to the Debye length alone cannot describe ion current rectification [3]: pore geometry and surface charges must also be taken into account. From the perspective of ion concentration polarization, this seems logical: the Debye length is independent of the absolute charge, while the main determining factor in ion concentration polarization is the difference in the currents carried by the counterions and the co-ions, which is related to the absolute charge. Therefore, we think the Debye length has been given too much consideration in nanopore research in the past. Moreover, at high concentrations, the concept of the Debye length as an electrical screening length is not physically meaningful anymore: for example, at 10 mM KCl, the Debye length is 3 nm, while this concentration corresponds to ≈ 1 ion/(4 nm)^3^: ions are discrete on the scale of the Debye length, while the Debye length is derived from a continuum description. 10 mM is at the upper limit of usability of the continuum description: at higher concentrations, activity coefficients may come into play due to interionic interactions. Conversely, concentrations lower than 10 mM would yield less than 20 ions in the uncharged nanopore, which also limits the applicability of the continuum description. This issue could be overcome by combining Brownian dynamics within the nanopore with the PNP equations outside [22]. The combination is expected to result in less effective screening of ions in the nanopore due to their discreteness and induced surface charges [23], leading to a lower conductance and higher selectivity.

In contrast to the simulations reported by Vlassiouk *et al*. [4], with I-V curves presenting a kink, the shape of the I-V curves after 0.2 *μ*s and at steady state shown here corresponds well to the shape of experimental I-V curves reported in [20, 21, 24, 25]. This is most likely due to the differences in boundary conditions. In our simulations, voltage equipotential lines inside the membrane are not perpendicular to the surface, and we observe ion concentration polarization outside the nanopore, while this part is not simulated when applying Donnan equilibrium conditions at both entrances of the nanopore. Simultaneous concentration increases and decreases in rectifying nanochannels have however previously been observed by Daiguji *et al*. (Fig 3a in [5]) and Kubeil *et al*. [3], who also simulated parts of the reservoirs, but these variations have not been identified as resulting from ion concentration polarization.

**Fig 13:**
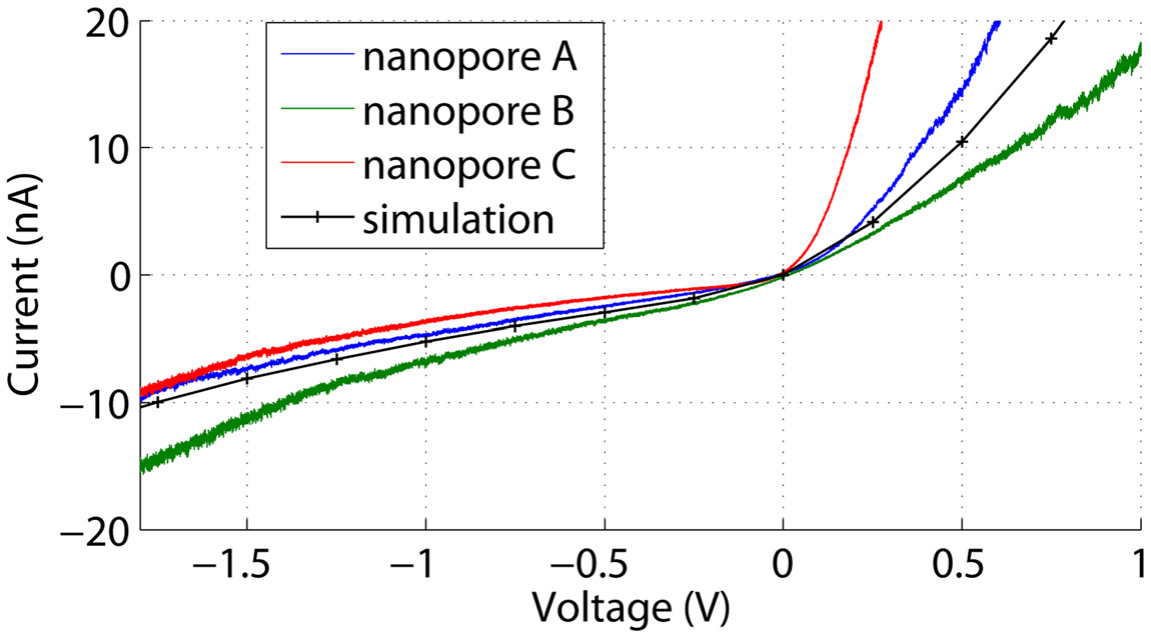
Current-voltage characteristics of 3 different nanopores and a corresponding simulation. The 3 nanopores present (A) *g*∣_0 V_ = 9 nS, (B) *g*∣_0 V_ = 11 nS, and (C) *g*∣_0 V_ = 17 nS within a buffer solution containing 100 mM KCl. Ion current rectification is observed as well as an overlimiting conductance in the low-conductance voltage polarity. The simulation represents a 10 nm nanopore in a 5 nm thick membrane with a surface charge configuration as in Fig 5c with a surface charge density of -100 mC/m^2^.

Fig 13 shows current-voltage characteristics of rectifying nanopores in silicon nitride membranes immersed in a buffer solution containing 100 mM KCl. Although these characteristics changed over time and could in some cases even change polarity, the major interesting feature in this figure is the overlimiting behaviour, which has to our knowledge not been observed before for a membrane containing a single nanopore. This overlimiting behaviour is well captured by a simulation of a 10 nm pore in a 5 nm thick membrane with a surface charge configuration as in Fig 5c and a surface charge density of -100 mC/m^2^. Since the nanopores have been formed with dielectric breakdown, their actual geometry and surface charge densities cannot readily be determined. Hence, the surface charge density and pore size in the simulation have been chosen to yield a realistic rectification ratio and absolute current values in combination with a nitride thickness that was chosen to reproduce the curvature of the overlimiting current. A thickness of 5 nm was found, which is realistic given that the KOH etch alone, performed on an unprocessed wafer, reduced the nitride thickness from about 22 nm to 8 to 9 nm, as has been measured with x-ray reflectometry. Hence, voltage-induced polarization charges can indeed explain the observed phenomenon.

### Influence of a conical shape

Similar surface charge configurations as in Fig 5 have been simulated for a conical nanopore with an opening angle of 20°, resulting in a largest opening with a diameter of 17 nm, which we positioned at the right. The corresponding figures are shown in S1 Supporting Information. In the case of uniform surface charges, the current at positive voltages is higher than at negative voltages, as previously observed by Siwy [25] for conical nanopores in symmetric electrolyte conditions. Ion concentration polarization mainly affects concentrations outside the nanopore at the entrance of the smallest pore opening, and within the nanopore instead of at the entrance of the largest pore opening. The fact that the nanopore conductance is mainly sensitive to concentrations within the pore explains the observed rectification. In our simulations, we also observed the effect of the increased conductance of the electric double-layer at more negative bias voltages, which results in a decreasing rectification ratio. This effect has not been observed in conical nanopores of several *μ*m long with a low opening angle due to the lower membrane capacitance. With our current simulation software however, such long pores cannot be simulated within a reasonable time frame, due to the large mesh required.

The configuration with surface charges only within the pore shows an opposite rectification: the current at positive voltages is lower than at negative voltages. At positive voltages, the depletion occurs at the smallest nanopore entrance, and the nanopore conductance is decreased more than at negative voltages, when the depletion occurs at the largest entrance.

In the past, several models for ion current rectification have been proposed [25]. Woermann modeled the increase or decrease within a conical nanopore, explicitly neglecting its counterpart outside the pore [26, 27], similarly to Cervera *et al*. [1], who used Donnan equilibrium boundary conditions at both entrances of the pore. Therefore, our model is more complete, as we applied boundary conditions further away from the pore. The model of Siwy however is different: it describes the low-conductance state as if counterions are trapped inside the nanopore. However, as this results in a local counterion concentration increase, the conductivity is locally increased instead of decreased, leading to a contradiction in the model. Only Wei *et al*. acknowledged the fact that concentration polarization occurs [28]. They studied a specific geometry in which a charge-selective nanopore was connected to two non-selective conical regions under a different angle, and attributed rectification to differences in limiting currents. Therefore, the model of Wei *et al*. corresponds to ours, but was applied to a nanopipet with an asymmetric geometry rather than a nanopore with asymmetric surface charges. It therefore seems that, except for Wei *et al*. in 1997, ion concentration polarization, resulting in both a concentration increase and decrease, has been systematically overlooked in the nanopore field, just as in the field of electrochromatography [29].

## Conclusion

In this work, we performed time-dependent and steady state simulations of ion currents through nanopores, using less restrictive boundary conditions than commonly assumed. These simulations revealed that, due to slow co-diffusion of K^+^ and Cl^−^, steady state is generally not reached in simulations, nor in practice. Furthermore, it has been shown that current-voltage characteristics are determined by the interplay of ion concentration polarization and the occurrence of polarization charges at the membrane in response to the applied voltage. Ion concentration polarization is responsible for the observed limiting conductances and ion current rectification in nanopores, while polarization charges at the membrane surface can explain the overlimiting conductances observed in simulations and measurements.

## Supporting Information

S1 Supporting Information Simulations of conical nanopores with different surface charge geometries, and the mesh size effect. (PDF)

## References

[1] Cervera J, Schiedt B, Neumann R, Mafe S, Ramirez P (2006) Ionic conduction, rectification, and selectivity in single conical nanopores. J Chem Phys 124 10.1063/1.2179797 16542096

[2] Wolfram MT, Burger M, Siwy ZS (2010) Mathematical modeling and simulation of nanopore blocking by precipitation. J Phys Condens Matter 22: 454101 10.1088/0953-8984/22/45/454101 21339589

[3] Kubeil C, Bund A (2011) The role of nanopore geometry for the rectification of ionic currents. J Phys Chem C 115: 7866–7873. 10.1021/jp111377h

[4] Vlassiouk I, Smirnov S, Siwy Z (2008) Nanofluidic ionic diodes. Comparison of analytical and numerical solutions. ACS Nano 2: 1589–1602. 19206361 10.1021/nn800306u

[5] Daiguji H, Yang P, Majumdar A (2004) Ion transport in nanofluidic channels. Nano Lett 4: 137–142. 10.1021/nl0348185

[6] Daiguji H, Oka Y, Shirono K (2005) Nanofluidic diode and bipolar transistor. Nano Lett 5: 2274–2280. 10.1021/nl051646y 16277467

[7] Stein D, Kruithof M, Dekker C (2004) Surface-charge-governed ion transport in nanofluidic channels. Phys Rev Lett 93: 035901 10.1103/PhysRevLett.93.035901 15323836

[8] Vlassiouk I, Smirnov S, Siwy Z (2008) Ionic selectivity of single nanochannels. Nano Lett 8: 1978–1985. 10.1021/nl800949k 18558784

[9] Gracheva ME, Vidal J, Leburton JP (2007) p-n semiconductor membrane for electrically tunable ion current rectification and filtering. Nano Lett 7: 1717–1722. 10.1021/nl0707104 17516680 PMC2553517

[10] Dimitrov V, Mirsaidov U, Wang D, Sorsch T, Mansfield W, et al (2010) Nanopores in solid-state membranes engineered for single molecule detection. Nanotechnology 21: 065502 10.1088/0957-4484/21/6/065502 20061599

[11] Hall JE (1975) Access resistance of a small circular pore. J Gen Physiol 66: 531–532. 10.1085/jgp.66.4.531 1181379 PMC2226214

[12] Rosenstein JK, Wanunu M, Merchant CA, Drndic M, Shepard KL (2012) Integrated nanopore sensing platform with sub-microsecond temporal resolution. Nat Methods 9: 487–492. 10.1038/nmeth.1932 22426489 PMC3648419

[13] Han A, Creus M, Schürmann G, Linder V, Ward TR, et al (2008) Label-free detection of single protein molecules and protein-protein interactions using synthetic nanopores. Anal Chem 80: 4651–4658. 10.1021/ac7025207 18470996

[14] Kwok H, Briggs K, Tabard-Cossa V (2014) Nanopore fabrication by controlled dielectric breakdown. PLoS One 9: e92880 10.1371/journal.pone.0092880 24658537 PMC3962464

[15] Kim SJ, Song YA, Han J (2010) Nanofluidic concentration devices for biomolecules utilizing ion concentration polarization: Theory, fabrication, and applications. Chem Soc Rev 39: 912–922. 10.1039/b822556g 20179814 PMC2929016

[16] Li G, Wang S, Byun CK, Wang X, Liu S (2009) A quantitative model to evaluate the ion-enrichment and ion-depletion effect at microchannel-nanochannel junctions. Anal Chim Acta 650: 214–220. 10.1016/j.aca.2009.07.044 19720195

[17] Chang HC, Yossifon G, Demekhin EA (2012) Nanoscale electrokinetics and microvortices: How microhydrodynamics affects nanofluidic ion flux. Annu Rev Fluid Mech 44: 401–426. 10.1146/annurev-fluid-120710-101046

[18] Kalman EB, Sudre O, Vlassiouk I, Siwy ZS (2009) Control of ionic transport through gated single conical nanopores. Anal Bioanal Chem 394: 413–419. 10.1007/s00216-008-2545-3 19089413

[19] Zangle TA, Mani A, Santiago JG (2010) Theory and experiments of concentration polarization and ion focusing at microchannel and nanochannel interfaces. Chem Soc Rev 39: 1014–1035. 10.1039/b902074h 20179822

[20] Schiedt B, Healy K, Morrison AP, Neumann R, Siwy Z (2005) Transport of ions and biomolecules through single asymmetric nanopores in polymer films. Nucl Instrum Methods Phys Res B 236: 109–116. 10.1016/j.nimb.2005.03.265

[21] White RJ, Zhang B, Daniel S, Tang JM, Ervin EN, et al (2006) Ionic conductivity of the aqueous layer separating a lipid bilayer membrane and a glass support. Langmuir 22: 10777–10783. 10.1021/la061457a 17129059

[22] van Oeffelen L, Van Roy W, Charlier D, Lagae L, Borghs G (2012) Modelling solid-state nanopores with a combination of the poisson-nernst-planck equations and brownian dynamics. In: Nanopores for Bioanalytical Applications: Proceedings of the International Conference, The Royal Society of Chemistry. pp. 51–56.

[23] Chung SH, Corry B (2005) Three computational methods for studying permeation, selectivity and dynamics in biological ion channels. Soft Matter 1: 417–427. 10.1039/b512455g 32646109

[24] Vlassiouk I, Siwy ZS (2007) Nanofluidic diode. Nano Lett 7: 552–556. 10.1021/nl062924b 17311462

[25] Siwy Z (2006) Ion-current rectification in nanopores and nanotubes with broken symmetry. Adv Funct Mater 16: 735–746. 10.1002/adfm.200500471

[26] Woermann D (2002) Analysis of non-ohmic electrical current-voltage characteristic of membranes carrying a single track-etched conical pore. Nucl Instrum Methods Phys Res B 194: 458–462. 10.1016/S0168-583X(02)00956-4

[27] Woermann D (2004) Electrochemical transport properties of a cone-shaped nanopore: Revisited. Phys Chem Chem Phys 6: 3130–3132. 10.1039/b316166h

[28] Wei C, Bard AJ, Feldberg SW (1997) Current rectification at quartz nanopipet electrodes. Anal Chem 69: 4627–4633. 10.1021/ac970551g

[29] Höltzel A, Tallarek U (2007) Ionic conductance of nanopores in microscale analysis systems: Where microfluidics meets nanofluidics. J Sep Sci 30: 1398–1419. 10.1002/jssc.200600427 17623420

