# Supplementary material for: Ion Current Rectification, Limiting and Overlimiting Conductances in Nanopores
Source: PLoS One. 2015 May 15;10(5):e0124171. doi: 10.1371/journal.pone.0124171 (PMC4433113; doi:10.1371/journal.pone.0124171)
Supplement: S1 Supporting Information — (PDF) [file pone.0124171.s001.pdf]

Supporting Information

This document presents the figures corresponding to the conical nanopore simulations, and the effect of the mesh size.

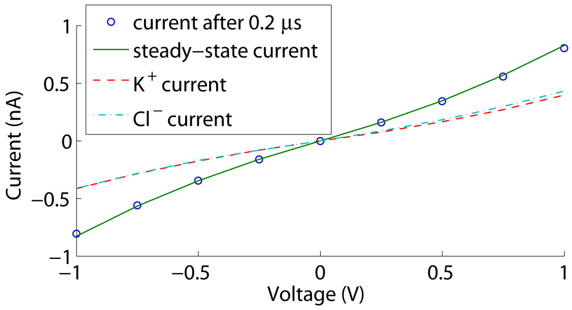

Fig. S1. Currents through an uncharged conical nanopore. The current after 0.2 μs is shown together with the steady state current and the K^+ and Cl^- contributions at steady state.

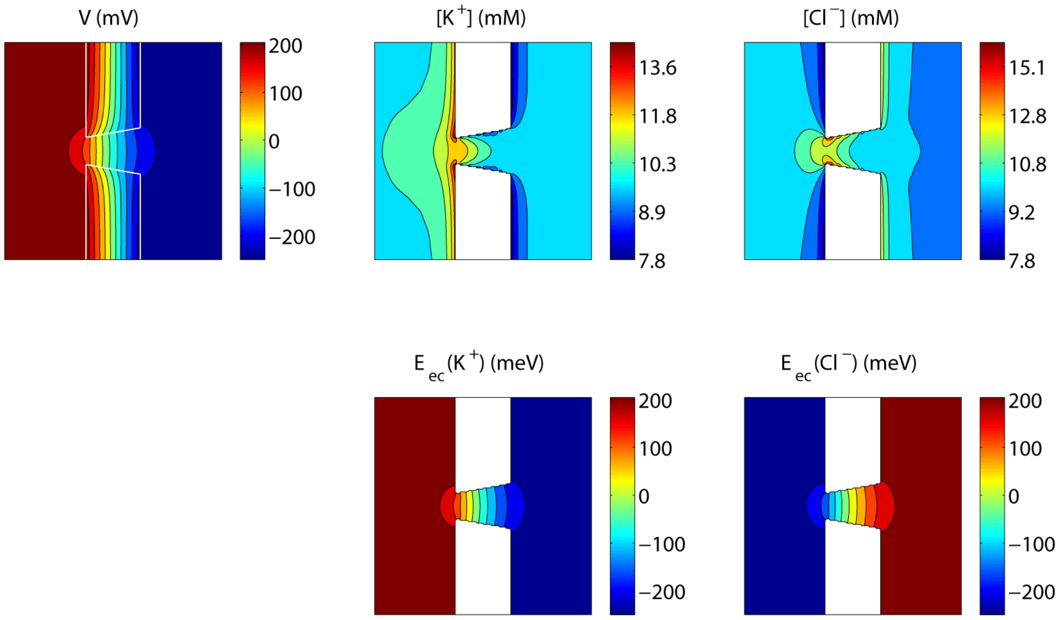

Fig. S2. Steady state voltage, concentrations and electrochemical potentials for an uncharged conical nanopore. The values shown are those in a plane through the axis of the nanopore, with $V_L - V_R = 0.5$ V.

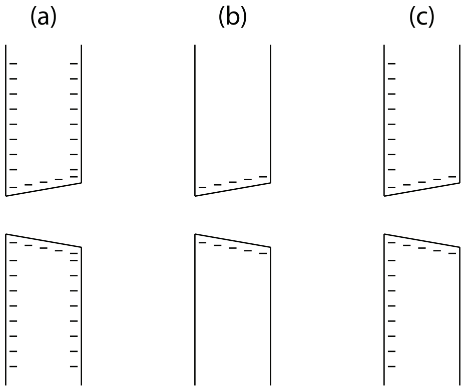

Fig. S3. Simulated surface charge configurations.

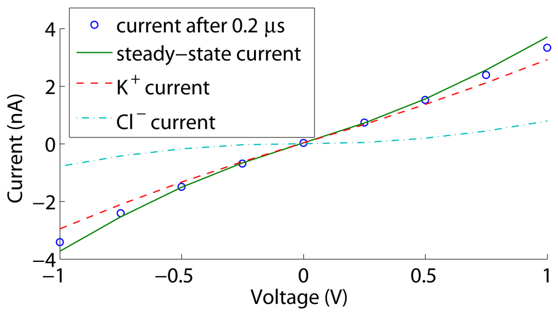

Fig. S4. Currents through the nanopore in Fig. S3a. The current after 0.2 μs is shown together with the steady state current and the K^+ and Cl^- contributions at steady state. The surface charge density is -50 mC/m^2.

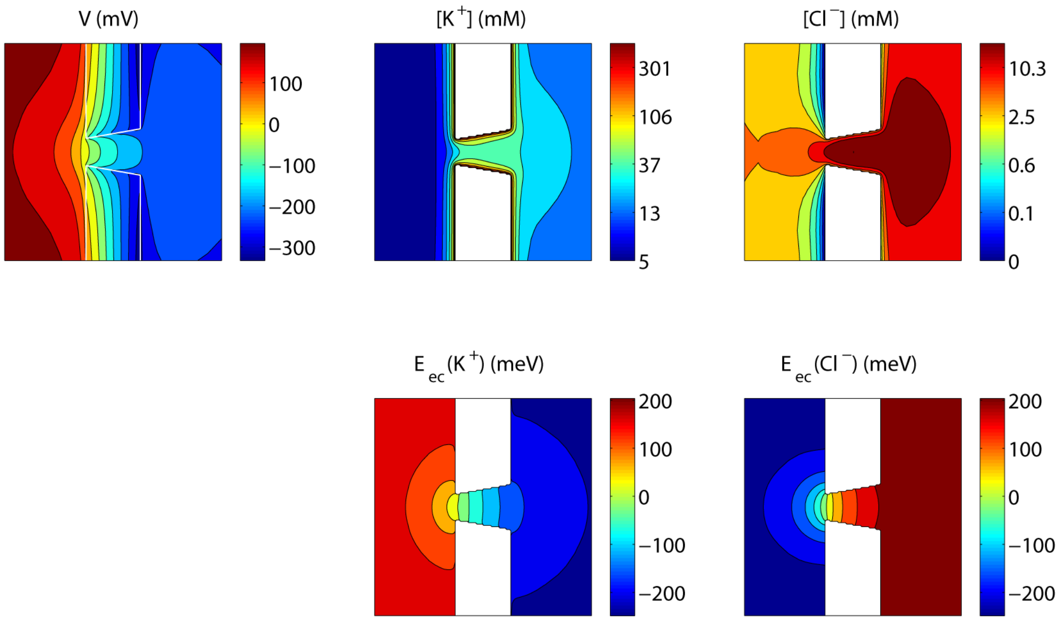

Fig. S5. Steady state voltage, concentrations and electrochemical potentials for the nanopore in Fig. S3a. The values shown are those in a plane through the axis of the nanopore, with $V_L - V_R = 0.5$ V and a surface charge density of -50 mC/m^2.

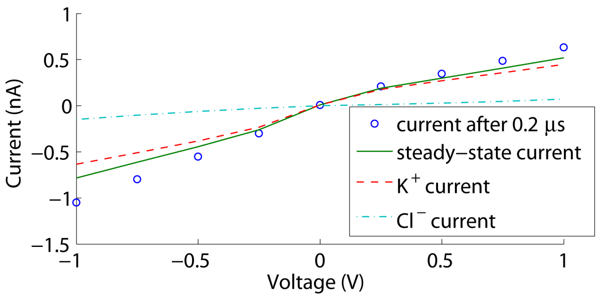

Fig. S6. Currents through the nanopore in Fig. S3b. The current after 0.2 μs is shown together with the steady state current and the K^+ and Cl^- contributions at steady state. The surface charge density is -50 mC/m^2.

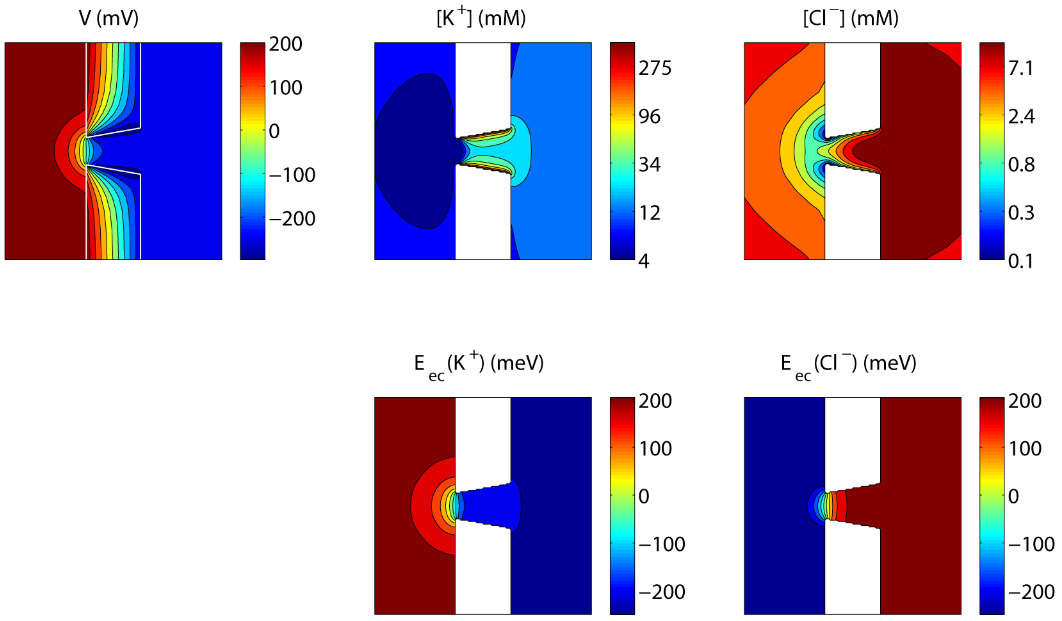

Fig. S7. Steady state voltage, concentrations and electrochemical potentials for the nanopore in Fig. S3b. The values shown are those in a plane through the axis of the nanopore, with $V_L - V_R = 0.5$ V and a surface charge density of -50 mC/m^2.

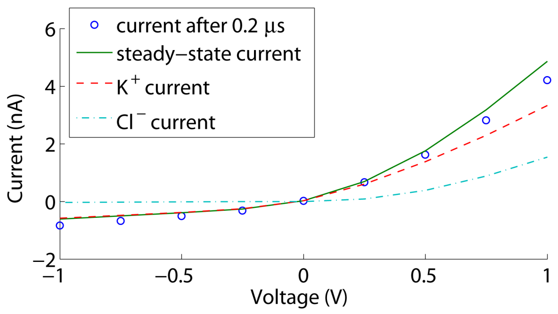

Fig. S8. Currents through the nanopore in Fig. S3c. The current after 0.2 μs is shown together with the steady state current and the K^+ and Cl^- contributions at steady state. The surface charge density is -50 mC/m^2.

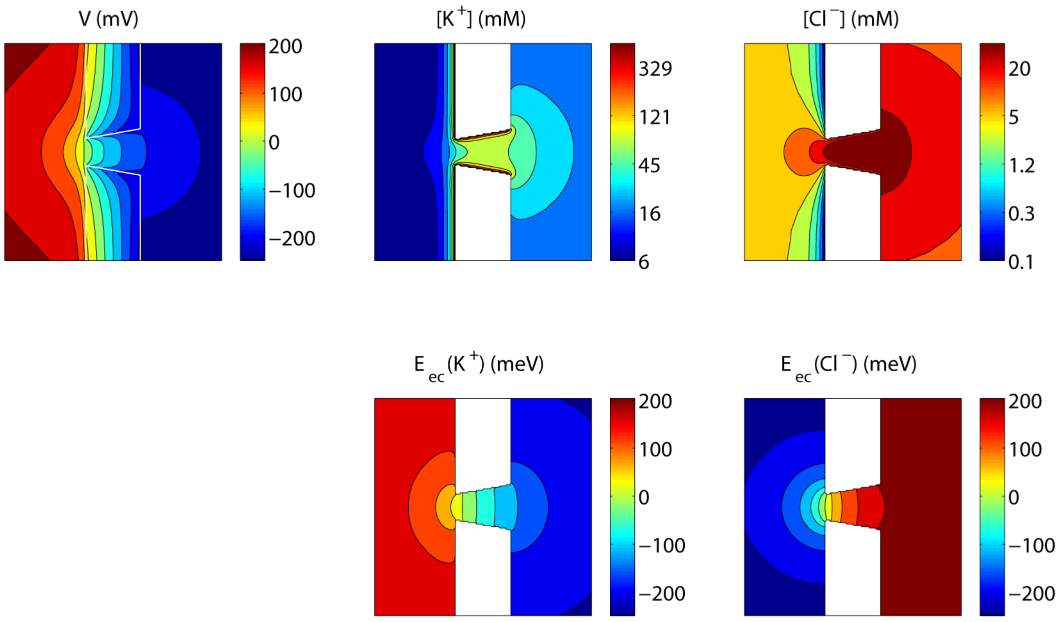

Fig. S9. Steady state voltage, concentrations and electrochemical potentials for the nanopore in Fig. S3c. The values shown are those in a plane through the axis of the nanopore, with $V_L - V_R = 0.5$ V and a surface charge density of -50 mC/m^2.

Fig. S10 shows the same simulation as Fig. 8 in the manuscript, with mesh sizes increased by a factor of 2. When comparing the two figures, the effect on the electrochemical equipotential lines at the membrane surface is clearly visible.

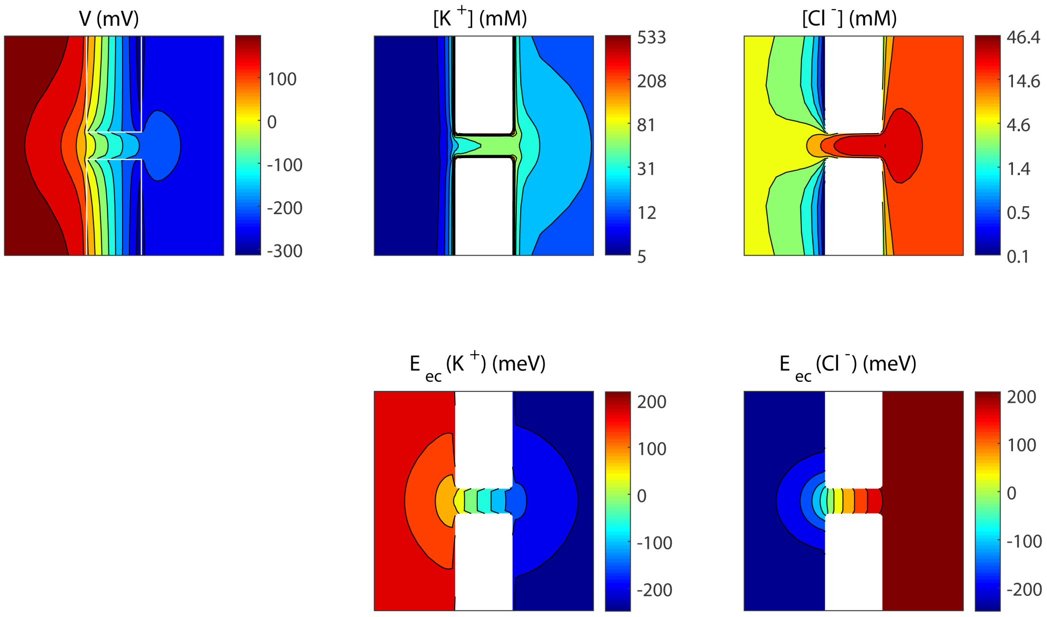

Fig. S10. Steady state voltage, concentrations and electrochemical potentials as in Fig. 8 of the manuscript, but with mesh sizes twice as large. Inside the nanopore, steps of 1 nm are used. Outside the pore, the steps in both coordinates increase with the distance to the pore along that coordinate.
